# Appendagite épiploïque primitive: à propos de cinq cas

**DOI:** 10.11604/pamj.2015.20.4.5509

**Published:** 2015-01-05

**Authors:** Houcine Maghrebi, Helmi Slama, Rachid Ksantini, Amine Makni, Fadhel Fteriche, Sofiene Ayadi, Wael Rebai, Amine Daghfous, Faouzi Chebbi, Adel Ammous, Mohamed Jouini, Montassar Kacem, Zoubeir Ben Safta

**Affiliations:** 1 Faculté El Manar, Faculté de Médecine de Tunis, Service de Chirurgie A, Hôpital La Rabta, Tunis, Tunisie

**Keywords:** Appendagites épiploïques, urgence, chirurgie, imagerie, epiploic appendagitis, emergency, surgery, imagery

## Abstract

La torsion de frange épiploïque (ou appendagite) est une pathologie rare qui survient principalement chez les adultes entre 20 et 50 ans. L'incidence de cette pathologie n'est pas réellement connue et elle varie de 2 à 7% chez les patients hospitalisés pour suspicion d'appendicite ou de sigmoïdite. Nous rapportons cinq cas d'appendagites dont nous précisons les particularités cliniques, radiologiques et thérapeutiques. Il s'agit de 5 patients dont l’âge moyen est de 34.6 ans (24-55). Le sexe ratio est de 1.5. Le principal motif de consultation était un syndrome douloureux de l'abdomen principalement au niveau de la fosse iliaque droite. L'examen abdominal montrait toujours une sensibilité localisée. La fièvre était présente chez 3 patients. Le bilan biologique révèle un syndrome inflammatoire biologique chez trois patients. Les examens complémentaires radiologiques en particulier échographie abdominale et TDM abdominale ont éliminé formellement une urgence chirurgicale et ont évoqué le diagnostic d'appendagite dans trois cas. Trois patients ont bénéficié d'une cœlioscopie diagnostique confirmant le diagnostic d'appendagite. L’évolution était favorable chez tous les patients. Les appendagites épiploïques primitives sont des étiologies rares et sous-estimées de syndrome abdominal aigu. Le diagnostic peut être affirmé par imagerie notamment avec le scanner hélicoïdal injecté, permettant d'instaurer ainsi un traitement médical premier et d’éviter un traitement chirurgical et des hospitalisations excessives.

## Introduction

La torsion de frange épiploïque (ou appendagite) est une pathologie rare qui survient principalement chez les adultes entre 20 et 50 ans. L'incidence de cette pathologie n'est pas réellement connue et elle varie de 2 à 7% chez les patients hospitalisés pour suspicion d'appendicite ou de sigmoïdite.

## Méthodes

Nous rapportons cinq cas d'appendagites colligés au service de chirurgie A de l'hôpital la Rabta. Plusieurs variables ont été étudiées: les particularités cliniques, radiologiques et thérapeutiques

## Résultats

**Figure 1:**
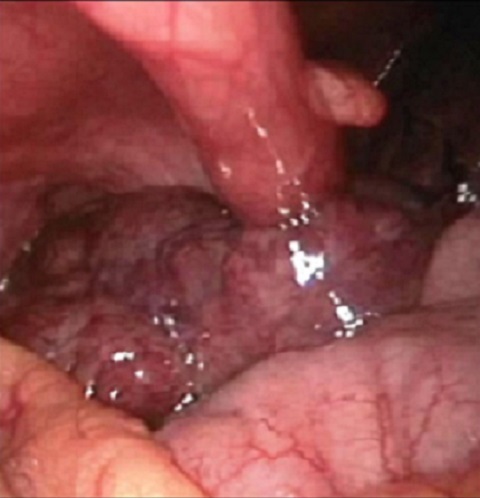
Vue per opératoire de la frange épiploïque nécrosée

**Figure 2:**
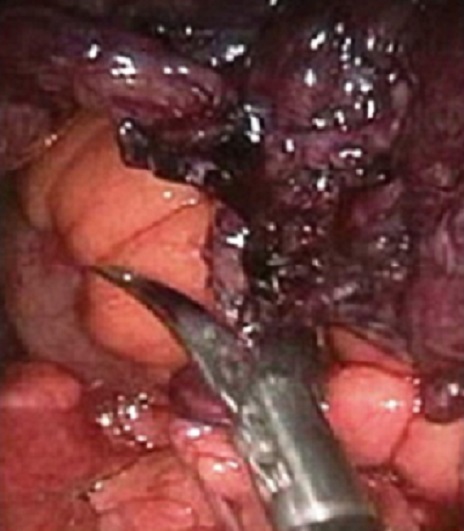
Vue per opératoire de la frange épiploïque nécrosée

**Observation 1**: Le premier patient est âgé de 28 ans, sans antécédents pathologiques particuliers. Il était admis en urgences pour des douleurs abdominales brutales, diffuses évoluant depuis 48h, maximales au niveau de la fosse iliaque droite associée à une fièvre. A l'admission, le patient ne présentait ni diarrhée ni hématurie. L'examen de l'abdomen trouvait une sensibilité au niveau de la fosse iliaque droite et en péri-ombilical. Le reste de l'examen clinique était sans anomalie. La biologie était sans anomalie notamment pas de syndrome inflammatoire. L’échographie abdominale était sans anomalie. Devant le doute sur une appendicite aiguë, une cœlioscopie diagnostique en urgence était décidée: il a été découvert en per opératoire un appendice épiploïque tordu et nécrosé (Figure 1, Figure 2). Le geste réalisé était un clippage du pédicule avec section de la frange épiploique nécrosée. Le patient était mis sortant à J2 post opératoire avec des suites opératoires simples.

**Figure 3:**
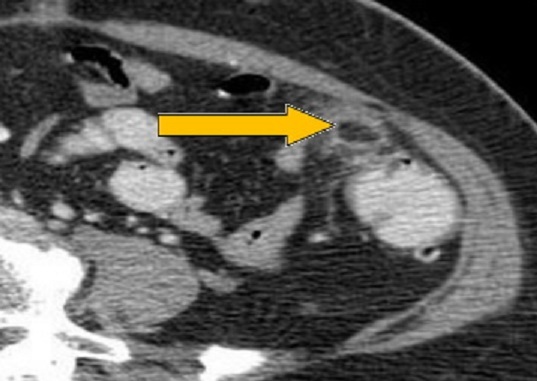
Aspect en tomodensitométrie de l'appendagite

**Observation 2**: Le deuxième patient est âgé de 24 ans. Il était admis en urgence pour un syndrome douloureux de la fosse iliaque gauche évoluant depuis 72 h, à type de crampe sans trouble du transit. A l'examen, il était apyrétique avec une défense de la fosse iliaque gauche. A la biologie il y avait une hyperleucocytose. L’échographie abdominale objectivait un épanchement intra-péritonéal de faible abondance. Le scanner abdominal (Figure 3) avait éliminé le diagnostic de diverticulite. Il existait par contre en amont du côlon gauche une zone hypodense ovalaire, de densité graisseuse de 2 cm de diamètre, limitée par un liseré hyperdense de quelques millimètres au contact de la paroi abdominale antérolatérale gauche. Devant la persistance des douleurs, le malade a été abordé par voie laparoscopique qui avait montré une frange épiploïque nécrosée. Une résection de cette dernière était réalisée. Les suites étaient simples et le patient a été mis sortant au deuxième jour postopératoire.

**Observation 3**: Il s'agit d'une patiente âgée de 29ans, non tarée qui consulte pour douleurs de la fosse iliaque droite évoluant depuis 24 heures sans signes urinaires associés. L'examen retrouve une patiente subfébrile à 37,8°C, une sensibilité nette de la fosse iliaque droite. A la biologie il existait un syndrome inflammatoire biologique avec GB à 13200 et une CRP à 39. La BHCG était négative. Nous avons porté le diagnostic d'une appendicite aiguë et avons abordé la patiente par voie laparoscopique. En per-opératoire l'appendice était sain, les annexes aussi et il existait une frange épiploïque au dépend du colon droit qui était inflammé, souffrante. Notre attitude était de respecter l'appendice et nous avons procédé à une exérèse de cette frange épiploïque. Les suites opératoires étaient simples.

**Observation 4**: Il s'agit d'un patient de 55 ans admis en urgence pour syndrome douloureux et fébrile de l'abdomen évoluant depuis quelques jours. L'examen abdominal montre une défense de la fosse iliaque droite. Le bilan biologique révèle un syndrome inflammatoire biologique. Les examens complémentaires radiologiques en particulier échographie abdominale et TDM abdominale ont éliminé formellement une urgence chirurgicale et ont confirmé le diagnostic d'appendagite. Un traitement médical était débuté à base d'anti-inflammatoires non stéroïdiens et d'antalgiques. L’évolution clinique et biologique était favorable et le patient était mis sortant au 7^ème^ jour, totalement asymptomatique. Le patient était revu à la consultation après 30 jours; son examen clinique et sa biologie étaient normaux.

**Figure 4:**
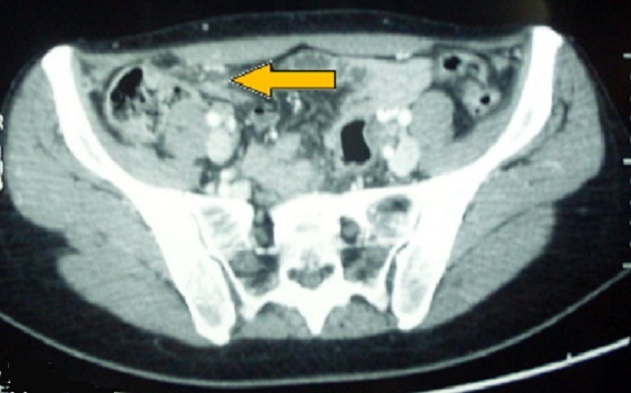
Aspect en tomodensitométrie de l'appendagite

**Observation 5**: Patiente âgée de 37 ans admise pour douleurs de la fosse iliaque droite évoluant depuis 5 jours, sans fièvre, ni vomissements, ni troubles du transit, ni signes urinaires. A l'examen on trouve une température à 37°C, une sensibilité de la fosse iliaque droite, le reste de l'abdomen était souple dépressible et indolore. A la biologie des GB à 7740, Hb à 9,7g/dl et une CRP à 3,4. L'ECBU était négatif et la BHCG négative. L’échographie abdominale était non concluante. Le scanner abdominal a objectivé une formation tissulaire ovalaire bien limitée au niveau de la fosse iliaque droite, à centre hypodense, hyperdense en périphérie, réalisant l'aspect de Ring Sign, associée à une densification de la graisse mésentérique avoisinante et évoquant une appendagite (Figure 4). La malade a été mise sous un traitement anti inflammatoire et antalgique. L’évolution a été marquée par l'amélioration de la symptomatologie au bout d'une semaine.

## Discussion

L'appendagite épiploïque primitive résulte d'une torsion avec ischémie ou thrombose veineuse spontanée d'un appendice épiploïque [1]. C'est dans un article de Lynn et al. [2] qu'apparaît en 1956 la première observation de cette entité. L'incidence de cette pathologie n'est pas réellement connue et elle varie de 2 à 7% chez les patients hospitalisés pour suspicion d'appendicite ou de sigmoïdite. Cependant la prévalence de l'appendagite est sous-évaluée, en raison du nombre de cas sous diagnostiqués. C'est une pathologie rare qui survient principalement chez les adultes entre 20 et 50 ans avec une légère prédominance masculine. Ces caractéristiques épidémiologiques sont retrouvées chez nos patients. Les appendices épiploïques correspondent à des formations graisseuses sous-péritonéales dont la longueur varie de 0.5 à 5 cm (moyenne de 3 cm). Ils sont répartis le long du cadre colique, et sont absents du rectum, leurs localisations sont, par ordre de fréquence, la charnière rectosigmoïdienne (57%), la région iléo-ccale (26%), le côlon ascendant (9%), le côlon transverse (6%) et le côlon descendant (2%). Dans notre série, la principale localisation était la fosse iliaque droite. Leurs fonctions physiologiques ne sont pas clairement définies [3, 4]. Leur vascularisation précaire et leur morphologie pédiculée les prédisposent à des phénomènes de torsion, d'ischémie et d'inflammation, phénomènes regroupés sous le nom d'appendagite. Le tableau clinique n'est pas spécifique. Cette pathologie se manifeste cliniquement par une douleur abdominale localisée, qui évoque souvent une appendicite iléo-cæcale ou une diverticulite. Les autres signes d'accompagnement comme les troubles du transit, les nausées, les vomissements ou la fièvre sont rares. La numération de formule sanguine montre parfois une hyperleucocytose modérée. D'autre part, devant cette symptomatologie, le diagnostic d'appendagite est rarement évoqué, d'où l'intérêt de l'imagerie. L’échographique montre un nodule graisseux (hyperéchogène cerné d'un liseré hypodense) sous-pariétal antérieur, au contact d'un côlon par ailleurs normal. La tomodensitométrie abdomino pelvienne avec injection de produite de contraste iodé est l'examen de référence dans l'appendagite. Elle peut permettre de faire le diagnostic en montrant un nodule hypodense adjacent à la paroi du côlon, avec un liseré périphérique hyperdense traduisant l'inflammation de la séreuse [4]. La cœlioscopie diagnostique est une alternative diagnostique lorsque la tomodensitométrie abdominale est contre indiquée, indisponible ou devant un doute diagnostic tel le cas de nos deux premiers malades. En effet, la cœlioscopie a actuellement une place reconnue, en diminuant la morbidité pariétale aux laparotomies et en raccourcissant la durée d'hospitalisation [5, 6]. Elle permet non seulement un diagnostic rapide sans multiplier l′imagerie, mais aussi de traiter la lésion en évitant une laparotomie dans bon nombre de cas comme l'illustre le cas de trois de nos patients. Le traitement de l'appendagite est toujours conservateur par antalgiques (paracétamol) et anti-inflammatoires durant une dizaine de jours. La symptomatologie se résout souvent spontanément en moins d'une semaine [7].

## Conclusion

Les appendagites épiploïques primitives sont des étiologies rares et sous-estimées de syndrome abdominal aigu. Le diagnostic peut être affirmé par imagerie notamment avec le scanner hélicoïdal injecté, permettant d'instaurer ainsi un traitement médical premier et d’éviter un traitement chirurgical et des hospitalisations excessives.
